# Port site tuberculosis a rare post operation entity: A case report

**DOI:** 10.1016/j.amsu.2022.103703

**Published:** 2022-04-29

**Authors:** Ibad-ur Rehman, Fareeha Farooqui, Sania Waseem, Ka Yiu Lee, Nadira Mamoon, Umar riaz, Dawood Tahir, Sehrish latif

**Affiliations:** a Shifa College of Medicine, Shifa Tameer e Millat University, Islamabad, Pakistan; b Shifa International Hospital, Islamabad, Pakistan; c Swedish Winter Sports Research Centre, Department of Health Sciences, Mid Sweden University, Östersund, Sweden

**Keywords:** Case report, Laparoscopy cholecystectomy, Port site infection, Tuberculosis

## Abstract

Laparoscopic cholecystectomy is one of the most common procedures done worldwide. Post-surgical site infections are common, yet there are occurrences of uncommon complications, including port site tuberculosis (TB).

We report a case of a 62-year-old man who was the victim of post-surgical site infection of port sites caused probably by improper sterilization. The patient lacked any common symptoms of tuberculosis and his initial lab investigations were not affirmative. A biopsy depicting the growth of multiple epithelioid granulomas finally led to the diagnosis of port site tuberculosis.

The patient was treated by incision and drainage followed by anti-tubercular therapy. This treatment regime showed complete resolution of disease on follow-ups.

Such cases require multidisciplinary team (Surgery, Pathology and Infectious disease department in our case) inputs for prompt diagnosis and treatment.

## Highlights

• In post-op patients of laparoscopic surgeries, even rare port site complications like port site tuberculosis can occur.
• Biopsy is the best way to confirm diagnosis of port site tuberculosis.
• Multidisciplinary approach is required for prompt diagnosis and treatment in such cases.

## 1 Introduction and importance

Postoperative surgical site infections are one of the most common entities encountered by surgeons in tertiary care hospitals [1].Post laparoscopic procedures port site infections are also not infrequent [2,3]. Compared to other procedures post-cholecystectomy port site infections and more specifically port site tuberculosis is a rare occurrence [4].Few investigations on port site tuberculosis are available to date.

In this case report, we present a 62-year-old male with no known comorbid who was operated on for cholecystitis in a local medical center in his hometown. The patient presented with port site infection and induration on right hypochondrium port. To the best of our knowledge, it is the first case being reported in an old man with no known comorbid.

This case report adheres to the SCARE criteria [5] and highlights the importance of proper sterilization methods like autoclaving which can help prevent port site infection.

## 2 Case presentation

Our case report entails a 62-year-old retired male of south Asian origin. Patient had no known allergies at the time of presentation and wasn't using any regular medications. The patient presented in the outpatient department with complaints of redness, pain, and itching around his wound at the port sites for the past 4 weeks.

The patient had undergone laparoscopic cholecystectomy one month back in a local medical center. Post-operation he complained of port site inflammation in the epigastric region accompanied by redness and itching. He also complained of irritation over the right hypochondrium port. He had no associated complaints of fever of any grade, headache, malaise, loss of appetite, cough with or without sputum nor did he notice any weight changes.

On examination, the patient was an average-sized man sitting in mild discomfort. The patient had port site wounds with redness around the epigastric region. The margin of the wounds was indurated, raised, and about 1cm in size. There was discharge of serous nature. The rest of his abdominal examination was normal. He had no swollen lymph nodes as well.

Initially, he was investigated for his wound infection with complete blood picture, pus cultures, AFB smear and he was prescribed Augmentin (625mg TDS), Paracetamol (1 g TDS) for treatment and symptomatic relief. He was advised to follow up with reports after 5 days.

**Fig. 1:**
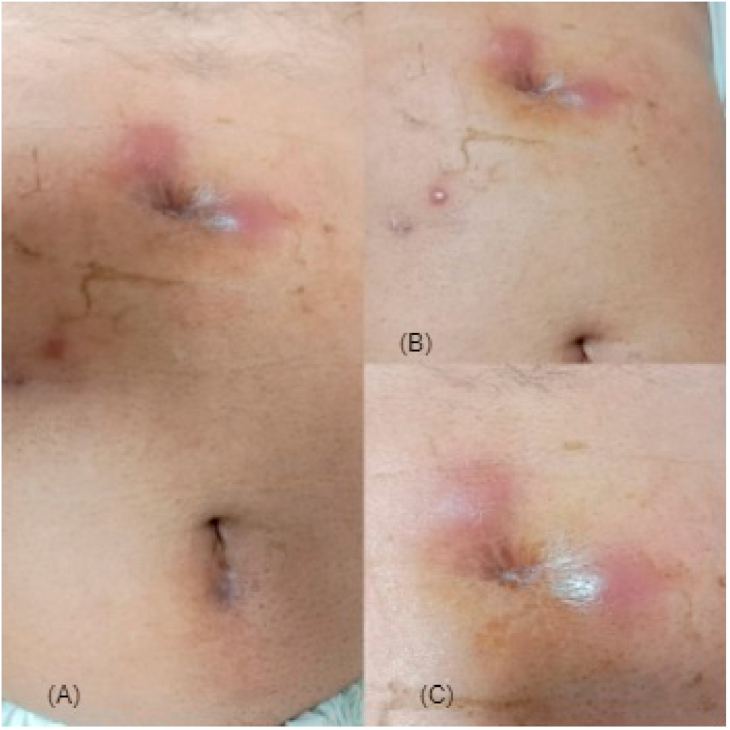
Section A, B and C showing port site infection and induration.

The patient returned two weeks later with an unsettled infection and his labs. His AFB smear was negative and pus culture showed moderate pus cells. Since his initial tests were not conclusive an MTB DNA along with port site biopsy was advised. Meanwhile, the patient was planned for incision and drainage under local anesthesia. The procedure was performed by Consultant General Surgeon in a minor operation theatre (OT) setting. Pus was drained and patient was relieved (see Fig. 1).

**Fig. 2:**
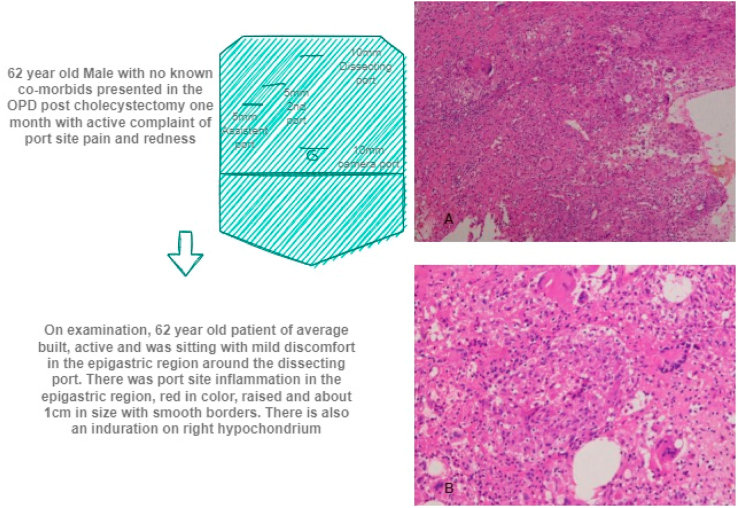
Brief summary of presentation of the patient along with pathology images A and B.

Histopathology of the infected tissue showed chronic granulomatous inflammation (Fig. 2). On microscopic examination multiple epithelioid granulomas with Langerhans giant cells were observed, suggesting tuberculosis. Definitive caseation was not seen. Also, MTB DNA was not detected.

The patient was transferred to the infectious disease department where he was started on anti-tubercular therapy. A 2-week follow-up showed how his infection was resolved with the therapy. This case report adheres to the SCARE criteria [5].

## 3 Clinical discussion

Laparoscopic Tuberculosis is a rare surgical complication. This case report aims to prevent such cases by identifying the loophole in sterilization and providing a tested treatment plan [4].If we study laparoscopic operations being conducted across the world, port site infections are quite common [1,2].Expecting a similar case in our patient, the first rational diagnosis was of port site infection and the patient was treated as such.

In our case, we suspected port site infection early and prompt treatment was provided to the patient but initial investigations including AFB and pus culture made the case relatively difficult to diagnose. Later on, the biopsy proved that the treatment opted was correct as proven by the patient's improvement and earlier reports [[1], [2], [3], [4]]. Our case included insights from the medicine, pathology, and infectious disease department making it a case of multidisciplinary interest.

It is suggested that when suspecting such a similar case in a patient following negative results in the initial lab investigations including blood culture, pus C/s and the usual, the sample should be sent to histopathology for prompt diagnosis. This suggestion stems from the fact that the previous reports also show how the diagnosis was confirmed on biopsy after initial negative results, as shown in Table 1 [[6], [7], [8], [9]].

**Table 1:** Comparison of cases reporting port site tuberculosis in patients of various demographics.

Cases reported	Patient (with relevant demographics)	Sterilization technique used	Investigations	Treatment plan followed
1)Ramesh H et al. [6]	67y/F/cholecystectomy	Not mentioned	Diagnosis on biopsy (caseating granulomas) Pus C/S did not show any growth)	Debridement (ineffective) Followed by Anti-TB therapy
2)Jagdish N, Sameer R, Omprakash R. [7]	48y/F/cholecystectomy	2% glutaraldehyde	Diagnosis on biopsy	Wide excision of sinus Followed by 4 drug anti-TB therapy
3)Chintamani, Kumar V, Singhal V [8]	65/F/cholecystectomy	Not mentioned	Diagnosis on biopsy Pus C/S positive	Treatment by excision Followed by Anti-Tb therapy
4)Faridi S, Siddiqui B, Singh K, Aslam M	28/F/cholecystectomy	Not mentioned	Diagnosis on biopsy Negative AFB	Anti-tubercular therapy

Our case was managed in a sequential way where the infection was treated first and then incision and drainage were performed. Previous reports show a similar pattern which includes drainage and excision apart from a minor change, we used empirical antibiotics in the patient initially [[6], [7], [8]].After the biopsy results, previous studies also show how anti-tubercular therapy was the best treatment with promising results and these drugs were given in our case as well [[6], [7], [8], [9]]. A multi-disciplinary effort is required to tackle such cases.

One of the limitations of this report stems from the patients’ lack of knowledge regarding his previous procedure. Therefore, we can only speculate that the source of infection was poorly sterilized instruments, which is supported by previous reports [[7], [8], [9]].

It does build up the case for the selective sterilization techniques for laparoscopic operations. These might help put a leash on the stern growth in post-op infections, especially in countries with poor sanitary conditions and facilities where infections are relatively more common and deadly.

It is also recommended to use more efficient sterilization and disinfectant techniques like autoclaving in order to tackle such surgical complications.

## 4 Conclusion

In a post laparoscopic patient, absence of common symptoms of Tuberculosis like fever, weight loss and malaise does not exclude a diagnosis of Port site tuberculosis. Proper sterilization is also required alongside disinfection to prevent any post operation infections in patients undergoing laparoscopic cholecystectomy.

## Ethical approval

We further confirm that any aspect of the work covered in this manuscript that has involved human patients has been conducted with the ethical approval of all relevant bodies and that such approvals are acknowledged within the manuscript. IRB approval was obtained. Written consent to publish potentially identifying information, such as details or the case and photographs, was obtained from the patient(s) or their legal guardian(s).

## Sources of funding

None to declare.

## Author contribution

IR, FF, SW, KYL,: conceived the idea, designed the study, drafted the manuscript and gave the final approval.

NM, UR, DT, SL: conducted literature search and created illustrations, revised the manuscript.

## Trial registry number

1. Name of the registry: NOT APPLICABLE
2. Unique Identifying number or registration ID:
3. Hyperlink to your specific registration (must be publicly accessible and will be checked):

## Guarantor

Ka Yiu Lee.

Swedish Winter Sports Research Center, Department of Health Sciences, Mid Sweden University, Östersund, Sweden. kyle.lee@miun.se.

Ibad ur Rehman.

Shifa college of Medicine, Shifa Tameer e Millat University, Islamabad, Pakistan. ibadrehmaan@gmail.com.

## Consent

Written informed consent was obtained from the patient for publication of this case report and accompanying images. A copy of the written consent is available for review by the Editor-in-Chief of this journal on request.

## Provenance and peer review

Not commissioned, externally peer-reviewed.

## Declaration of competing interest

None to declare.
